# Resting State Dynamic Functional Connectivity in Neurodegenerative Conditions: A Review of Magnetic Resonance Imaging Findings

**DOI:** 10.3389/fnins.2019.00657

**Published:** 2019-06-20

**Authors:** Massimo Filippi, Edoardo G. Spinelli, Camilla Cividini, Federica Agosta

**Affiliations:** ^1^Neuroimaging Research Unit, Institute of Experimental Neurology, Division of Neuroscience, IRCCS San Raffaele Scientific Institute, Milan, Italy; ^2^Neurology Unit, IRCCS San Raffaele Scientific Institute, Milan, Italy; ^3^Vita-Salute San Raffaele University, Milan, Italy

**Keywords:** dynamic functional connectivity, fMRI, neurodegeneration, dementia, Alzheimer’s disease, Parkinson’s disease, Lewy bodies, frontotemporal dementia

## Abstract

In the last few decades, brain functional connectivity (FC) has been extensively assessed using resting-state functional magnetic resonance imaging (RS-fMRI), which is able to identify temporally correlated brain regions known as RS functional networks. Fundamental insights into the pathophysiology of several neurodegenerative conditions have been provided by studies in this field. However, most of these studies are based on the assumption of temporal stationarity of RS functional networks, despite recent evidence suggests that the spatial patterns of RS networks may change periodically over the time of an fMRI scan acquisition. For this reason, dynamic functional connectivity (dFC) analysis has been recently implemented and proposed in order to consider the temporal fluctuations of FC. These approaches hold promise to provide fundamental information for the identification of pathophysiological and diagnostic markers in the vast field of neurodegenerative diseases. This review summarizes the main currently available approaches for dFC analysis and reports their recent applications for the assessment of the most common neurodegenerative conditions, including Alzheimer’s disease, Parkinson’s disease, dementia with Lewy bodies, and frontotemporal dementia. Critical state-of-the-art findings, limitations, and future perspectives regarding the analysis of dFC in these diseases are provided from both a clinical and a technical point of view.

## Introduction: From Static to Dynamic Functional Connectivity

Neurodegenerative diseases are characterized by a progressive loss of neurons associated with deposition of aberrant proteins, leading to alterations of the structural and functional properties of the brain (Kovacs, 2017). In the last decades, the non-invasive resting-state (RS) functional magnetic resonance imaging (RS-fMRI) technique has been widely applied in these clinical populations, to investigate more in depth the spatial topology and strength of interactions between brain networks (Smitha et al., 2017; Hohenfeld et al., 2018).

Functional magnetic resonance imaging uses the blood-oxygenation-level dependent (BOLD) signal, which is sensitive to spontaneous neural activity. In particular, low-frequency oscillations (<0.1 Hz) of the BOLD signal are analyzed to obtain functional information of brain networks. Functional connectivity (FC) quantifies the temporal correlation of functional activation in different brain regions and can be expressed in terms of pairwise Pearson’s correlation coefficients, covariance, or mutual information between time series, revealing specific networks (Smitha et al., 2017). FC has been recognized as an important biomarker for better understanding the pathophysiological mechanisms of numerous neurodegenerative diseases, including Alzheimer’s disease (AD) (Filippi et al., 2017), Parkinson’s disease (PD) (Baggio et al., 2015; Filippi et al., 2019), and frontotemporal dementia (FTD) (Filippi et al., 2017).

So far, the implicit hypothesis on which FC analysis has been based is the assumption of temporal stationarity of the functional interaction between connections. Considering the dynamic nature of brain activity, a novel approach is provided by dynamic functional connectivity (dFC), which considers the temporal fluctuations of functional connections in faster timescales (Hutchison et al., 2013). Unlike conventional static FC, which is obtained from the correlation within an entire time series, dFC refers to the brain activity within sub-portions of time series (Menon and Krishnamurthy, 2019). Major efforts have been made to identify and analyze time-varying, but recurring, FC sub-patterns of coupling among brain regions, constituting the brain “chronnectome” (Calhoun et al., 2014).

The aim of this review is to describe the contribution of dFC studies in RS conditions for a better understanding of neurodegenerative diseases. We are going to focus on the most common approaches to analyze dFC and review recent findings in this field concerning AD, PD and other parkinsonisms, and FTD. We conclude this work summarizing caveats, limitations and future perspectives regarding dFC analysis.

## Methodological Overview

Several computational strategies have been implemented to characterize temporal and spatial variations of BOLD signal (Wee et al., 2016; Jie et al., 2018; Liu et al., 2018). The most common approach is provided by the sliding-window technique (Chen et al., 2016, 2017; de Vos et al., 2018; Diez-Cirarda et al., 2018), characterized by the selection of a time window – shorter than the whole-scan time – whose data points are used to calculate FC metrics. The window is shifted in time by a fixed number of data points, referred as step, which defines the overlap between two successive windows. The step duration ranges from one single data point to the length of the window (i.e., non-overlapping windows) (Jie et al., 2018; Park et al., 2018).

In combination with the sliding-window approach, several studies have applied clustering methods to identify reproducible, transient patterns and to evaluate the commonly used graph metrics, the dwell time, defined as the number of consecutive windows in a specific state, and the number of transitions between states (Allen et al., 2014).

**FIGURE 1:**
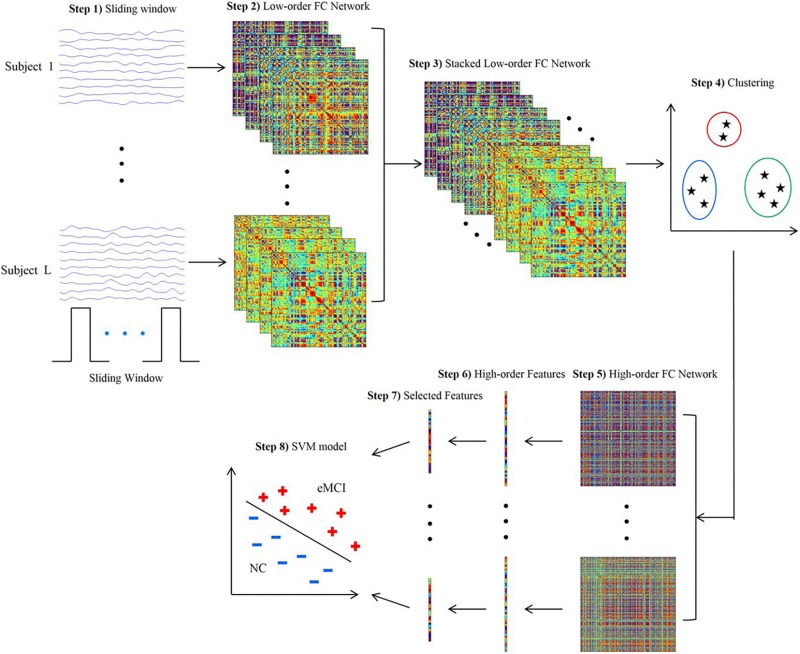
Framework for construction of high-order functional connectivity (FC) network. (1) Partition of the RS-fMRI time series into multiple overlapping segments of subseries applying sliding-window technique; (2) collection of low-order FC matrices, one for each subseries; (3) stack of all matrices of all subjects together to obtain correlation time series for each element; (4) application of the clustering algorithm to group all the correlation time series; (5) construction of high-order FC network, considering the mean correlation time series for each cluster as vertex and the pairwise Pearson’s correlation coefficient between each pair of vertices as weight; (6) calculation of local clustering coefficients; (7) selection of a discriminative feature subset from the local clustering coefficients; (8) implementation of support vector machine (SVM) model for classification. RS-fMRI, resting-state functional magnetic resonance imaging; FC, functional connectivity (reproduced with permission from Chen et al., 2016).

Since a subject could be in more than one state at a given point, the concept of “meta-states” and meta-state measures has been introduced to intuitively characterize the dynamic fluidity in FC (Miller et al., 2016; Premi et al., 2019). Meta-state measures include the number of occupied meta-states, number of switches between meta-states, greatest distance between two meta-states and overall distance (Premi et al., 2019). Furthermore, the sliding-window approach can be integrated with the application of independent component analysis (ICA) to identify spatial maps in the windowed BOLD signal and assess variability or graph theoretical metrics (Jones et al., 2012; de Vos et al., 2018; Premi et al., 2019). The sliding-window approach can also be used jointly with classification algorithms to exploit the information resulting from patterns of dFC (Chen et al., 2016; Guo et al., 2017; Figure 1).

Alternative approaches to evaluate dFC are represented by time-frequency analysis, dynamic connectivity regression (DCR) and dynamic connectivity detection (DCD), which are data-driven techniques for detecting FC change points within RS fMRI time series, and derive dynamic information from such points (Xu and Lindquist, 2015).

## Alzheimer’s Disease

Alzheimer’s disease is the most common neurodegenerative cause of dementia (Alzheimer’s, 2016) and has been extensively studied by means of advanced MRI techniques. The inclusion of RS fMRI into imaging protocols in AD has been particularly advantageous, as the difficulty to obtain subjects’ cooperation could influence task-related fMRI results. Conspicuous evidence has shown decreased FC of the default mode network (DMN) across the AD continuum, including patients with full-blown AD dementia and amnestic mild cognitive impairment (MCI) (Greicius et al., 2004; Bai et al., 2008; Agosta et al., 2012; Koch et al., 2012). Decreased connectivity within the DMN – consisting of the posterior cingulate, inferior parietal, inferolateral temporal, anterior cingulate, prefrontal, and hippocampal regions – is often accompanied by increased connectivity in the attentional fronto-parietal and salience networks, likely mirroring compensatory mechanisms (Agosta et al., 2012; Badhwar et al., 2017). Disconnection between posterior (i.e., posterior cingulate and parietal regions) and anterior DMN nodes (i.e., anterior cingulate and prefrontal regions) was found to cause relative decreased connectivity within the posterior DMN and increased connectivity within the anterior DMN (Jones et al., 2011). Functional rearrangements have demonstrated clinical usefulness for predicting conversion to AD in MCI patients (Bai et al., 2011; Petrella et al., 2011; Li et al., 2016).

Given the high consistency of these findings across static FC studies, AD represents a good candidate to apply dFC approaches to the field of neurodegenerative disorders, since capturing the evolving architecture of brain networks over short periods of time might provide further pathophysiological insights into these conditions, and eventually better diagnostic or prognostic indicators. The first study investigating dFC of AD patients examined changes over time of a modularity metric using a sliding-window analysis (Jones et al., 2012). The non-stationary nature of the brain modular organization was demonstrated and related with significant variations of the dwell time within different sub-network configurations of the DMN in subjects with AD dementia compared with healthy controls; specifically, AD patients spent less time in brain functional states with strong posterior DMN region contribution and more time in states with greater anterior DMN region contribution (Jones et al., 2012). A subsequent study investigated the evolution of dFC disruptions across the AD spectrum, showing alterations in patients with dementia compared to MCI and subjective cognitive decline (SCD) in terms of local dFC within the temporal, frontal-superior and default-mode networks; decreased global metastability between functional states was also found, supporting the hypothesis that oscillatory patterns are progressively altered over the AD continuum, eventually leading to a shrinkage of the “dynamic repertoire” (i.e., a smaller set of functional configurations) in the brain of AD patients (Cordova-Palomera et al., 2017). Consistently, another study showed a progressive loss of whole-brain metastability according to the severity of cognitive impairment along the AD continuum, reaching statistical significance only in patients with dementia, when compared with healthy controls (Demirtas et al., 2017).

Researchers have also aimed to identify dFC alterations that may represent candidate non-invasive diagnostic biomarkers in the early stages of AD. A sparse temporal network-based framework has been tested for the classification of patients with early MCI by means of support vector machine (SVM) algorithms, yielding an accuracy of approximately 80% in the discrimination from healthy controls, compared with accuracies ranging 62–72% using FC static approaches (Wee et al., 2016). Another recent study aimed to integrate both temporal and spatial properties of dFC networks for the classification of early and late MCI patients (Jie et al., 2018). Accuracies of approximately 78% were obtained when SVM algorithms were trained based on the dFC patterns of components of the DMN and temporal and cerebellar regions (Jie et al., 2018). An attempt to combine multiple dFC parameters for an automated classification of early MCI patients was recently proposed by applying a tensor model of spatio-temporal BOLD signal to each voxel of the white matter (WM), to be integrated with the information provided by the FC of grey matter (GM) regions (Chen et al., 2017). Such combined GM and WM approach yielded an accuracy of almost 79% in discriminating early MCI patients from healthy subjects, compared with 74% provided by GM dFC measures alone (Chen et al., 2017). Another promising approach is the clustering of standard-correlation time series for all pairs of brain regions (i.e., the classic “low-order” FC networks) into a smaller set of “high-order” dFC networks according to their intrinsic common patterns. The combination of high-order dFC with the conventional low-order analysis allowed SVM-based discrimination of early MCI patients from healthy subjects with an accuracy of 88%, outperforming other previous approaches (Chen et al., 2016; Figure 1).

## Parkinson’s Disease

Parkinson’s disease (PD) is the second most common neurodegenerative disorder and is characterized by dopamine depletion in the nigro-striatal system leading to progressive functional impairment (Poewe et al., 2017). Widespread functional rearrangements related to the development of motor and non-motor symptoms occur over the clinical progression in PD patients (Filippi et al., 2019). Several RS fMRI studies have identified alterations of the cerebello-thalamo-cortical circuit as a key hallmark of PD (Helmich et al., 2010; Hacker et al., 2012; Agosta et al., 2014; Akram et al., 2017), with reduced activation of the posterior putamen correlating with motor impairment as the most consistent finding (Herz et al., 2014). Furthermore, disrupted FC in the DMN, fronto-parietal, salience and associative visual networks has been linked to the development of cognitive deficits in PD (Amboni et al., 2015; Baggio et al., 2015; Putcha et al., 2015; Zhan et al., 2018). Particularly, normal decoupling between the DMN and fronto-parietal networks was reduced in PD patients with MCI (PD-MCI) (Baggio et al., 2015; Putcha et al., 2015), and FC alterations within the DMN were able to predict subsequent cognitive decline in cognitively unimpaired PD patients (van Eimeren et al., 2009; Tessitore et al., 2012; Putcha et al., 2015).

**FIGURE 2:**
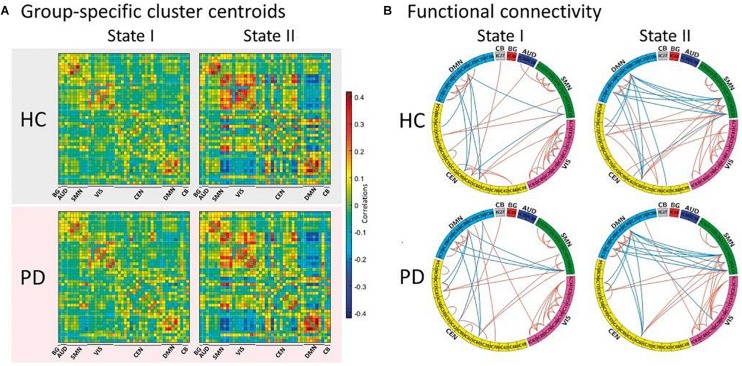
Functional connectivity state results. **(A)** Group-specific cluster centroid for each state, averaged across subject-specific median cluster centroids of each group [percentage of total occurrences for stage I and II: 83.4% and 16.6% in the healthy controls (HC) and 70.8% and 29.2% in the Parkinson’s disease (PD) group, respectively]. **(B)** Functional connectivity in each state is shown for healthy controls and Parkinson’s disease groups, representing the 5% of the functional connectivity network with the strongest connections. BG, basal ganglia; AUD, auditory; SMN, sensorimotor; VIS, visual; CEN, cognitive executive; CB, cerebellar network (reproduced with permission from Kim et al., 2017).

The first study assessing the dynamic functional properties of RS networks in PD patients identified two main FC configurations: a more frequent and strongly segregated state (defined as “State I”) and a less frequent, more integrated “State II” (Kim et al., 2017). Compared with healthy subjects, PD patients showed a significant decrease of dwell time in State I, with a proportional increase of dwell time in State II that was correlated with the severity of motor symptoms, indicating that the loss of functional segregation between brain networks might represent a key element in PD pathogenesis (Kim et al., 2017; Figure 2). To eliminate the possible influence of long-term dopaminergic therapy, dFC alterations were also assessed in early stage, drug-naïve PD patients, who showed decreased switching rate between dynamic states correlating with disease severity, supporting the view that a limited dynamic range of whole-brain FC might represent an early PD marker (Cordes et al., 2018; Zhuang et al., 2018). Another recent study focused on the spatial configuration of dFC alterations within two homogeneous subunits of the putamen of PD subjects, demonstrating a degradation of subregional specificity between the anterior and posterior putaminal subunits that was related with disease severity (Liu et al., 2018).

Along with motor impairment, dFC has also provided significant information regarding the underpinnings of cognitive symptoms in PD. Decreased dwell time in a more segregated state and increased state transitions have been demonstrated in PD-MCI patients compared with healthy controls, configuring a pattern that PD patients with normal cognition lacked (Diez-Cirarda et al., 2018). Increased dFC within the dorsal-attention network was found to predict attention performance in PD patients (Madhyastha et al., 2015), whereas a positive correlation between dFC of the DMN and performance on a visuospatial memory task has also been recently reported (Engels et al., 2018).

## Other Neurodegenerative Conditions

To date, most dFC studies assessing patients with neurodegenerative conditions have focused on the two most common diseases, i.e., AD and PD. Considering also the novelty of these approaches, evidence regarding other pathological entities is currently scarce, but in rapid development.

Dementia with Lewy bodies (DLB) is among the most common causes of dementia after AD, and is characterized by cognitive fluctuations, parkinsonism, and visual hallucinations (McKeith et al., 2017). Classic RS-fMRI static studies have shown FC reductions in widespread brain networks in DLB subjects, with desynchronization of cortical and subcortical areas within the attention-executive networks correlating with cognitive fluctuations (Lowther et al., 2014; Peraza et al., 2014). Considering the transient nature of some of the main features of DLB (i.e., cognitive fluctuations and hallucinations), dFC studies are expected to provide fundamental insights into the pathophysiology of this disease. Indeed, dFC has demonstrated significant differences in DLB patients compared with healthy subjects in visual (i.e., the occipito-parieto-frontal and medial occipital networks) and attentional networks (i.e., the right fronto-parietal control network), which also showed decreased mutual dependency, suggesting that temporal disconnection between these networks might be relevant for DLB pathogenesis (Sourty et al., 2016).

Frontotemporal dementia is another frequent neurodegenerative condition encompassing a wide range of clinical presentations, including behavioral, executive, language and motor deficits (Olney et al., 2017). To our knowledge, no study has assessed dFC alterations in patients with FTD. However, a recent work focused on presymptomatic carriers of FTD-causing mutations (Premi et al., 2019), being FTD an inherited autosomal disorder in 30–40% of cases (Rohrer and Warren, 2011). Mutation carriers showed lower number of meta-states, decreased switching rate between meta-states and shorter meta-state total distance compared with healthy controls, demonstrating that a reduced dynamic fluidity and restricted dynamic range in brain functional “chronnectome” is an early event in the development of FTD (Premi et al., 2019). The assessment of such alterations in the phase which is closest to clinical conversion might be a promising research field for the development of biomarkers to be used in clinical trials of FTD.

## Caveats, Limitations and Future Directions

Based on the evidence here reviewed, dFC studies have shed new light on the pathophysiological alterations underlying the most common neurodegenerative diseases. However, important concerns remain regarding the possible influence of vigilance fluctuations during the fMRI scan. Although patients are routinely instructed to stay awake for the whole scan duration, sleep disturbances are frequent clinical features of dementias and parkinsonian syndromes (Malhotra, 2018), and fluctuating alertness will affect FC (Tagliazucchi et al., 2012; Haimovici et al., 2017). This important issue would be overcome by simultaneous EEG-fMRI acquisition (Tagliazucchi and Laufs, 2014; Allen et al., 2018), although this approach is technically challenging and has not been explored in neurodegenerative conditions yet.

Some technical caveats also need to be considered. The sliding-window technique has been repeatedly applied because of its analytical simplicity and easy implementation: in most studies, the number of BOLD signal subseries K is decided based on the window length W, the number of temporal image volumes N and the step, according to the formulation K = [(N – W) / s] +1 (Chen et al., 2016; Wee et al., 2016; Guo et al., 2017). However, the window length, as well as the step parameter, are matter of debate: choosing a short window could increase the risk of misleading spurious fluctuations, while choosing a long window could fail to identify state transitions (Preti et al., 2017). A trade-off must be reached: at present, different studies suggested a window length of 30–60 s as sufficient for detecting dFC changes (Jones et al., 2012; Diez-Cirarda et al., 2018; Premi et al., 2019), even though some studies opted for longer windows (Chen et al., 2016; Wee et al., 2016). An alternative way to find the optimum window length is represented by the time-frequency analysis (Cordes et al., 2018). The step parameter is also chosen arbitrarily, commonly ranging one to two volumes for overlapping windows (Quevenco et al., 2017; Liu et al., 2018), although a few studies adopted non-overlapping windows (Jie et al., 2018; Park et al., 2018). Another crucial point is the choice of window shape: rectangular, modulated rectangular or tapered windows are the most used (Diez-Cirarda et al., 2018; Premi et al., 2019).

Beyond these methodological aspects, there are still some unsolved questions regarding dFC. Neurobiological underpinnings and mechanisms of dynamic states have to be clarified (Smith et al., 2011), as the possibility that reported changes may be driven by signal noise or sampling variability needs to be considered (Laumann et al., 2017). Indeed, the fluctuations in the sliding-window correlation time series can be associated to dFC or simply generated by random noise, so more complex statistical models are required to deal with this issue (Hindriks et al., 2016). The artifact problem that applies to conventional resting-state fMRI is also a crucial aspect for dFC analysis (Nalci et al., 2019), as the BOLD signal is sensitive to non-stationary physiological processes, such as head motion (Power et al., 2014) and blood partial pressure of carbon dioxide (pCO2) due to respiration (Power et al., 2018). Moreover, important factors to take into account for a correct interpretation of dFC results are the selection of the *a priori* atlas or ICA algorithm used to obtain regions of interest and the assessment of specific FC metrics. Finally, the reliability and reproducibility of dFC patterns are still a challenge, although some efforts have been made on solving this issue (Abrol et al., 2017).

## Conclusion

Assessing dFC is a promising way to better understand neurodegenerative processes and investigate novel disease diagnostic and prognostic biomarkers. However, future developments are needed to rule out the influence of vigilance fluctuations, overcome the limitations of the sliding-window approach – possibly using other methods as time-frequency analysis –, identify the most informative dFC metrics, and minimize artifacts by means of adequate preprocessing, so as to be more confident in the description and interpretation of these findings.

## Author Contributions

MF contributed to the study concept and acted as study supervisor. All authors contributed to writing, reading, and approving the final version of the manuscript.

## Conflict of Interest Statement

MF is Editor-in-Chief of the Journal of Neurology; has received compensation for consulting services and/or speaking activities from Biogen Idec, Merck-Serono, Novartis, Teva Pharmaceutical Industries; and has received research support from Biogen Idec, Merck-Serono, Novartis, Teva Pharmaceutical Industries, Roche, Italian Ministry of Health, Fondazione Italiana Sclerosi Multipla, and ARiSLA (Fondazione Italiana di Ricerca per la SLA). FA is Section Editor of NeuroImage: Clinical; has received speaker honoraria from Biogen Idec and Novartis; and receives or has received research supports from the Italian Ministry of Health, AriSLA (Fondazione Italiana di Ricerca per la SLA), and the European Research Council. The remaining authors declare that the research was conducted in the absence of any commercial or financial relationships that could be construed as a potential conflict of interest.
